# The Initial Treatment Plan Versus the Actually Performed Treatment of Patients With Temporomandibular Disorders in the First 6 Months After the Initial Visit

**DOI:** 10.1111/joor.70012

**Published:** 2025-07-01

**Authors:** Kaylee van Ee, Magdalini Thymi, Naichuan Su, Michail Koutris, Thiprawee Chattrattrai, Frank Lobbezoo

**Affiliations:** ^1^ Department of Orofacial Pain and Dysfunction, Academic Centre for Dentistry Amsterdam (ACTA) University of Amsterdam and Vrije Universiteit Amsterdam Amsterdam the Netherlands; ^2^ Department of Oral Public Health, Academic Centre for Dentistry Amsterdam (ACTA) University of Amsterdam and Vrije Universiteit Amsterdam Amsterdam the Netherlands; ^3^ Department of Masticatory Science Faculty of Dentistry, Mahidol University Bangkok Thailand; ^4^ Department of Orofacial Pain and Jaw Function, Faculty of Odontology Malmö University Malmö Sweden

**Keywords:** awake bruxism, sleep bruxism, temporomandibular joint disorders, treatment

## Abstract

**Background:**

Temporomandibular disorders (TMD) can impact on daily life, and are therefore important to treat with a fitting therapy. However, the factors that may influence the received treatment remain unknown.

**Objectives:**

To investigate the deviation between the indicated and received TMD treatment, and to identify patient and clinician characteristics that could influence the received treatment and the deviation from indicated treatment.

**Methods:**

This retrospective cohort study collected data on the indicated and received treatment of 140 TMD patients. The treatment modalities were counselling, occlusal appliance (OA), physical therapy, psychological treatment, contingent electrical stimulation, ecological momentary assessment and medication. Potential predictors for receiving treatment and deviation from indicated treatment included patient‐related factors such as TMD diagnosis, bruxism, psychosocial factors and clinician‐related factors such as clinician's specialty level and experience.

**Results:**

A good to perfect agreement between indicated and received treatments was observed for all treatment (84.3%–100%), except psychological treatment (66.4%). Received OA was associated with having a pain diagnosis (OR [95% CI] = 2.596 [1.189, 5.669], *p* = 0.017). In addition, received physical therapy was significantly associated with a pain diagnosis (OR [95% CI] = 3.876 [1.401, 10.721], *p* = 0.009), awake bruxism (OR [95% CI] = 1.730 (1.112, 2.690), *p* = 0.015) and clinician's level–being staff (OR [95% CI] = 6.068 [1.729, 20.553], *p* = 0.016). Received psychological therapy was significantly associated with a pain diagnosis (OR [95% CI] = 4.013 [1.077, 14.951], *p* = 0.038), sleep bruxism (OR [95% CI] = 1.381 [1.041, 1.830], *p* = 0.025), and physical symptoms (OR [95% CI] = 2.578 [1.561, 4.259], *p* < 0.001). No significant predictors were found for deviation.

**Conclusion:**

Receiving TMD treatment was associated with both patient‐related factors—such as a TMD diagnosis, bruxism and physical symptoms—and clinician‐related factors, such as the clinician's level of experience.

## Graphical abstract

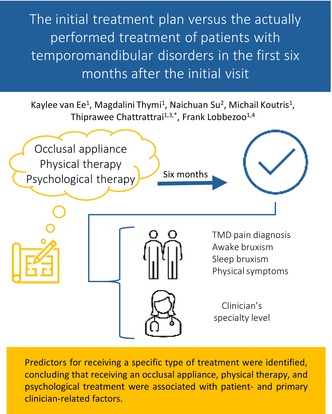

## 1 Introduction

Temporomandibular disorders (TMDs) are a group of conditions related to the masticatory muscles and/or temporomandibular joint (TMJ) [1]. The main reason for TMD patients to seek help is the presence of pain [2]. Besides pain, TMD patients can suffer from functional limitations as well, both muscle and joint‐related. As such, they can experience impaired movement, as well as clicking and/or crepitus TMJ sounds during mandibular movements. TMDs might have a negative impact on daily life, and are therefore important to understand and treat with a fitting therapy [3, 4, 5]. Moreover, non‐painful symptoms in the orofacial region, like tiredness, tension or stiffness, have been described as risk factors for future onset of TMD pain, and further investigation of their role within the TMD phenotype has been recommended [6, 7]. If present, it can be hypothesised that these symptoms could require treatment, even in the absence of pain.

The management of TMD includes multidisciplinary approaches, such as physical treatment and psychological treatment [8, 9]. It is known that multiple factors can influence the indicated treatment plan at baseline. Su et al. showed that different treatment plans were indicated for different types of patients characteristics and clinical examination [8]. Another study focused on clinician characteristics, and found that female examiners are more likely to indicate TMD management than male examiners, whereas years passed since graduation were associated with less TMD management indication [10]. Thus, clinician's characteristics may also influence the treatment indication of TMD patients.

The management of TMD can be a time‐consuming and costly process, and accurate prediction of the course of treatment will allow effective use of resources for both the patient and the clinician. However, to the best of the authors' knowledge, no studies exist on the topic of whether treatment indicated at baseline eventually changes as time passes and which factors influence this change. This study therefore aimed to investigate the deviation between the treatment indicated at baseline and the treatment that TMD patients actually received, as well as to identify patient and clinician characteristics that could potentially influence the actual received treatment and the deviation from indicated treatment.

## 2 Materials and Methods

### 2.1 Participants

The study was approved by the ACTA Ethics Committee (ref. No. 2021‐47 483), and followed the principles of the Declaration of Helsinki.

This retrospective cohort study was performed at the Clinic for Orofacial Pain and Dysfunction (OPD) of the Academic Centre for Dentistry Amsterdam (ACTA), Amsterdam, The Netherlands. Patients are referred to this clinic for orofacial pain and/or dysfunction complaints, and undergo a thorough diagnostic procedure at baseline. All patients who were referred to the OPD Clinic of ACTA between September 2020 and March 2021, with a follow‐up period of maximally 6 months from intake, were eligible to enrol the study.

The inclusion criteria were: aged 18 years and older; signed the informed consent form; having undergone the complete clinical examination of the Diagnostic Criteria for Temporomandibular Disorders (DC/TMD); having received a TMD pain and/or dysfunction diagnosis or a clinical diagnosis of non‐painful symptoms of the masticatory system; and having received a treatment indication at baseline. Potential participants' medical and/or dental condition did not affect whether participants were included or not.

### 2.2 Study Procedure

All patients completed a set of diagnostic questionnaires prior the first visit to the clinic. During the first visit, an oral history (anamnesis) and a comprehensive clinical examination according the DC/TMD were performed to establish a DC/TMD diagnosis. Moreover, patients who presented with non‐painful symptoms of the masticatory system, such as tiredness, tension or stiffness; who required treatment for these symptoms; and in whom these symptoms could be provoked during the DC/TMD clinical examination, received a clinical diagnosis of non‐painful symptoms. After receiving a diagnosis, an appropriate treatment plan was designed for each patient.

At the OPD clinic of ACTA, patients suffering from TMD can receive various management modalities to alleviate their complaints, provided by a multidisciplinary team of clinicians. These treatment modalities include counselling, occlusal appliance therapy, physical therapy, psychological therapy and myofeedback treatment. Additionally, ecological momentary assessment (EMA) treatment for awake bruxism (BruxApp smartphone application), contingent electrical stimulation (CES) for sleep bruxism (GrindCare) and analgesic medication may be prescribed [8, 9].

The type of treatment indicated at baseline was proposed during the intake by the clinician who conducted the examination. This recommendation was based on the anamnesis, clinical test results, the patients' specific signs, symptoms and preferences, as well as on the clinician's expertise and experience, and was thoroughly discussed with the patient.

The treatment included in the treatment plan at baseline was defined as ‘indicated treatment’. The clinical notes of each visit were reviewed. If any of the visits involved providing one of the above‐mentioned treatments within 6 months from baseline, that specific treatment was defined as ‘received treatment’.

To identify variables in clinician profiles, clinicians who performed the intakes received a consent form for the anonymous use of their personal data in this study. This data was only used when a clinician actively gave consent and filled in the clinician questionnaire completely. When no response was given, the data was listed as ‘missing data’.

The TMD diagnosis and entire treatment process were mapped based on the notes that the clinicians entered into the electronic health record (EHR), and the necessary data were entered in Castor, a web‐based electronic data capture software (Ciwit B.V., Amsterdam, The Netherlands).

### 2.3 Outcomes

#### 2.3.1 Types of Treatment

The types of treatment that could be indicated and performed include counselling, occlusal appliances, physical therapy, psychological treatment, contingent electrical stimulation (CES), EMA and medication. More information is provided below.

##### 2.3.1.1 Counselling

Counselling is defined as receiving information at baseline about the established diagnosis and/or receiving information at baseline about the aetiology of the complaints. Besides, patients can receive advice about the treatment course and be reassured about their complaints and diagnosis at baseline.

##### 2.3.1.2 Occlusal Appliance

This treatment can be used when sleep bruxism was reported. It consists of receiving a new occlusal appliance or of being advised to wear a pre‐existing occlusal appliance. In addition, the treatment includes (possible) follow‐ups at the OPD clinic. All appointments which involved receiving information about the new or pre‐existing occlusal appliances and/or any adjustments were included in the occlusal appliance treatment category.

##### 2.3.1.3 Physical Therapy

Physical therapy includes performing and/or instructing patients in physical exercises and relaxation therapy, provided by a dentist and/or a physical therapist. It also encompasses myofeedback therapy under the supervision of a physical therapist. Additionally, physical therapy for TMD complaints provided by an external physiotherapist was included in this treatment category.

##### 2.3.1.4 Psychological Treatment

Psychological treatment includes participating in a stress workshop provided by a psychologist at the OPD clinic, receiving individual psychological treatment in real life and/or through online sessions, and attending potential follow‐ups at the OPD clinic or externally.

##### 2.3.1.5 Contingent Electrical Stimulation (CES)

CES can be used for patients with sleep bruxism and is performed with an ambulatory device called GrindCare [11, 12]. This portable device helps patients gain insight into their overnight muscle activity through data recorded in a smartphone app. Besides its diagnostic purpose, GrindCare also provides a therapeutic intervention through contingent electrical stimulation—electrical impulses aimed at suppressing masticatory muscle activity [11, 12]. CES treatment involves the use of the GrindCare device for diagnostic and/or therapeutic purposes, including instructions on its use.

##### 2.3.1.6 Ecological Momentary Assessment (EMA)

EMA can be used to obtain real time reports of patients' complaints, behaviours or feelings. Patients are asked to answer the questions about their activities and experiences at fixed or random time points using a smartphone app called BruxApp [13]. EMA treatment includes the use of BruxApp for diagnostic and/or therapeutic purposes along with instructions on its use.

##### 2.3.1.7 Medication

This additional treatment involves the prescription or specific instruction for over‐the‐counter (pain) medication to support TMD management and/or to reduce complaints. This category includes paracetamol, NSAIDs, botox or other types of medication.

#### 2.3.2 Deviation From Indicated Treatment and Treatment Intensity

Deviation from the indicated treatment was considered when the indicated treatment and the received treatment did not fully match, that is, if an indicated treatment was not received and/or if a received treatment was not originally indicated. If it was unclear from the notes in the EHR whether a treatment was actually received, it was noted as a missing value.

### 2.4 Predictors

The potential predictors for the received treatment were selected based on previous research and consensus among the investigators, and were categorised in patient‐related factors and clinician‐related factors [8, 10, 14]. Patient‐related factors consists of data from questionnaires that were filled in by the partcipants before the intake appointment and the clinical examination based on the DC/TMD ultimately provided the established TMD diagnosis, including stretch of assisted mouth opening [15]. These factors included sex, age, diagnosis of TMD (non‐pain, pain), pain intensity and pain‐related impairment from Graded Chronic Pain Scale (GCPS), sleep bruxism from Oral Behaviours Checklist (OBC) and awake bruxism from OBC, anxiety from Generalised Anxiety Disorder‐7 (GAD‐7), physical symptoms from Patient Health Questionnaire‐15 (PHQ‐15), depression from Patient Health Questionnaire‐9 (PHQ‐9), Perceived stress from Perceived Stress Scale‐4 (PSS‐4), Optimism about effect of treatment from Life Orientation Test‐Revised (LOT‐R), previous TMD treatment (no, yes) and stretch of assisted mouth opening (< 5 mm, ≥ 5 mm) [16, 17, 18, 19, 20, 21]. Primary clinician‐related factors included sex, age (< 30, ≥ 30 years old), clinician's specialty level (trainee, staff) and experience since graduation (< 10, ≥ 10 years). All independent variables are presented in Table 1.

**TABLE 1:** Explanation of different scores of patient‐ and primary clinician‐related factors.

Independent variable	Score		Additional information
Sex	Female; male		—
Age	At least 18 years‐old		—
Diagnosis of TMD	TMD non‐pain diagnosis: DC/TMD joint disorder and non‐painful symptom diagnosis; TMD Pain diagnosis: DC/TMD pain, DC/TMD pain and joint disorder		A diagnosis based on the DC/TMD and clinical symptoms. The non‐painful symptoms diagnosis group is based on participants who did not fullfil the criteria for a pain and/or joint disorder diagnosis based on the DC/TMD, but did have complaints that indicated treatment need
Graded Chronic Pain Scale (GCPS)	0: No pain; 1: Low intensity pain; without disability; 2: High intensity pain; without disability; 3: Moderately limiting; 4: Severely limiting		A scale that measures characteristic pain intensity (CPI) for current pain, worst and mean pain in the past 30 days, and a point system to measure interference in daily, social and work activities, and days with interference due to pain
Oral Behaviours Checklist (OBC)	*Sleep bruxism*	*Awake bruxism*	Grinding or clenching teeth during sleep (sleep bruxism) and clenching, grinding and holding, tightening, or tensing muscles without clenching or bringing teeth together (OBC items 3–7) during wakefulness (awake bruxism) were assessed. Patients reported the frequency of sleep bruxism and awake bruxism activity over the past month. The highest score from OBC items 3–7 was used to score awake bruxism for the purposes of analyses
	0: none of the time; 1: < 1 night per month; 2: 1–3 nights per month; 3: 1–3 nights per week; 4: 4–7 nights per week	0: none of the time; 1: a little of the time; 2: some of the time; 3: most of the time; 4: all of the time	
7‐item Generalised Anxiety Disorder (GAD‐7)	0: none; 1: mild anxiety; 2: moderate anxiety; 3: severe anxiety		Questionnaire used to evaluate patients' anxiety during the last 2 weeks. Sum scores of 5, 10 and 15 represent cut‐points for mild, moderate and severe anxiety, respectively
15‐item Patient Health Questionnaire (PHQ‐15)	0: none ohysical symptoms; 1: low physical symptoms; 2: medium physical symptoms; 3: high physical symptoms		Physical symptoms are rated based on the frequency patients were bothered by any of these symptoms during the last 4 weeks. Sum scores of 5, 10 and 15 represent cut‐points for low, medium, and high physical symptoms, respectively
9‐item Patient Health Questionnaire (PHQ‐9)	0: none; 1: mild depression; 2: moderate depression; 3: severe depression		Questionnaire used to score patients' depression during the last 2 weeks. Sum scores of 5, 10, 15 and 20 represent cut‐points for mild, moderate, moderately severe, and severe depression, respectively
Perceived Stress Scale‐4 (PSS‐4)	Ranged from 0 to 4		Questionnaire used to score stress in the last 4 weeks. The higher scores indicated more stress. Sum scores of 0–16 was computed into 0–4
Life Orientation Test‐Revised (LOT‐R)	Ranged from 0: complaints cannot be helped, to 10; complaints can be resolved completely		Optimism about the effect of treatment on reduction of the complaints was measured by asking the question: ‘How optimistic are you that the complaints you have registered with can be solved?’
Previous TMD treatment	No; yes		—
Passive stretch of assisted mouth opening	< 5 mm; ≥ 5 mm		The distance between the front teeth of the lower and upper jaw is measured and noted. The clinician asks the patient to relax and assists whilst opening the mouth by performing outwards pressure with thumb and index finger on the patients' front teeth to reach a maximum assisted mouth opening
Clinician's sex	Female; Male		—
Clinician's age	< 30; ≥ 30 years old		—
Clinician's specialty level	Trainee; Staff		Primary clinicians' differentiation was divided into two groups, that is, staff (specialised in TMD and Orofacial Pain) or in training (not yet specialised in TMD and Orofacial Pain)
Clinician's experience since graduation	< 10; ≥ 10 years		

### 2.5 Statistical Analysis

To investigate the deviation from the indicated treatment, the agreement between indicated and received treatments was tested as percentage (%) for each specific type of treatment separately. Since no standardised cut‐off values exists for percentage agreement, this study defined the following thresholds: ≥ 60% as moderate or fair agreement, ≥ 80% as acceptable agreement, ≥ 90% as good agreement and 100% as perfect agreement.

To identify patient and clinician characteristics that could potentially influence the actually received treatment and the deviation from indicated treatment, first, the bivariate association of each potential predictor with each received treatment and deviation from indicated treatment was tested. The chi‐square test or Fisher's Exact test was used for dichotomous predictors. The Kolmogorov–Smirnov test was used to check for normality for continuous variables, and thereafter, the independent samples *T* test or Mann–Whitney *U* test was used for the continuous predictors. In addition, the Mann–Whitney *U* test was used to determine associations between ordinal predictors and the outcomes. Second, predictors with a univariate *p* value ≤ 0.20 were selected for inclusion in the subsequent multivariable binary logistic regression analyses with backward selection, based on previous research [9]. In the regression model, ordinal variables were entered as continuous variables. Statistical significance was set at *p* < 0.05.

In the present study, there was no predefined sample size. Statistical analysis was performed using IBM SPSS Statistics 28 software (IBM Corp., Armonk, New York, USA).

## 3 Results

### 3.1 Sample

A total of 229 patients visited the OPD Clinic at ACTA for an intake appointment between September 2020 and March 2021. Since not all of these intakes met the inclusion criteria for this study, the final sample consisted of 140 participants, of whom 106 (75.7%) were female. The median (Q1–Q3) age of the total sample was 40 (27.25–55.75) years. The majority of patients received a combination of treatments. Only 49 out of 140 patients received counselling alone or a combination of counselling with another treatment, as follows: 17 patients received counselling alone, nine patients received counselling with occlusal appliance, 20 patients received counselling with physical therapy, 2 patients received counselling with psychological treatment and 1 patient received counselling with contingent electrical stimulation.

### 3.2 Deviation From the Indicated Treatment

Table 2 shows that counselling was indicated and performed for all 140 participants. Meanwhile, CES, EMA and medication were indicated in very few patients. Deviations between the indicated and received treatments were observed in more than half of the participants (*n* = 80, 57.1%). There was a perfect agreement (100%) between indicated and received treatment for counselling. Good agreement between indicated and received treatments was observed for medication (97.1%) and CES (92.1%). Good agreement was observed for EMA (89.3%), occlusal appliance (85%) and physical therapy (84.3%). Fair agreement between indicated and received treatments was observed for psychological treatment (66.4%).

**TABLE 2:** Associations between indicated and actually received treatment per treatment category.

Indicated treatment	Received treatment		Agreement %
	No	Yes	
Counselling			
No	0	0	100
Yes	0	140	
Occlusal appliance			
No	29	4	85.0
Yes	17	90	
Physical therapy			
No	12	4	84.3
Yes	18	106	
Psychological treatment			
No	58	0	66.4
Yes	47	35	
CES			
No	126	2	92.1
Yes	9	3	
EMA			
No	124	4	89.3
Yes	11	1	
Medication			
No	135	3	97.1
Yes	1	1	

Abbreviations: CES, contingent electrical stimulation; EMA, ecological momentary assessment.

### 3.3 Patient and Clinician Characteristics associated with the Deviation of Treatment

Since counselling was indicated and received by all of the 140 participants, no statistical analyses were performed for this treatment category. Due to low counts, the treatment categories CES, EMA and medication were excluded from statistical analysis as well. Descriptive data for all potential predictor variables are presented in Table 3.

**TABLE 3:** Description of variables and univariate analyses (*n* = 140).

Factors (min–max)	Total sample (%) (*n* = 140)	Type of performed treatment									Deviation from indicated treatment		
		Occlusal appliance			Physical therapy			Psychological treatment					
		No (%) (*n* = 46)	Yes (%) (*n* = 94)	*p*	No (%) (*n* = 30)	Yes (%) (*n* = 110)	*p*	No (%) (*n* = 105)	Yes (%) (*n* = 35)	*p*	No (%) (*n* = 60)	Yes (%) (*n* = 80)	*p*
**Patient‐related factors**													
Sex				0.943^a^			**0.192** ^**a**^			0.495^a^			0.569^a^
Female	106 (75.7)	35 (76.1)	71 (75.5)		20 (66.7)	86 (78.2)		78 (74.3)	28 (80.0)		44 (73.3)	62 (77.5)	
Male	34 (24.3)	11 (23.9)	23 (24.5)		10 (33.3)	24 (21.8)		27 (25.7)	7 (20.0)		16 (26.7)	18 (22.5)	
Age													
(18–79)	40 (27.25–55.75)	43.5 (26.‐58.25)	39 (28–53.5)	0.624^c^	40 (24.5|‐59.25)	40 (28–53.5)	0.955^c^	38 (27–56)	45 (29–53)	0.580^c^	45.5 (29–56)	38 (26.25–51.75)	**0.176** ^**c**^
Diagnosis				**0.017** ^**a**^			**0.057** ^**a**^			**0.006** ^**a**^			0.956^a^
Non‐pain	37 (26.4)	18 (39.1)	19 (20.2)		12 (40)	25 (22.7)		34 (32.4)	3 (8.6)		16 (26.7)	21 (26.3)	
Pain	103 (73.6)	28 (60.9)	75 (79.8)		18 (60)	85 (77.3)		71 (67.6)	32 (91.4)		44 (73.3)	59 (73.8)	
GCPS													
(0–4)	1 (1–3)	1 (0–2)	2 (1–3)	**0.036** ^**c**^	1 (0–2.25)	2 (1–3)	**0.185** ^**c**^	1 (1–2)	2 (1–4)	**0.003** ^**c**^	1 (1–3)	2 (1–2.75)	0.790^c^
Missing	1		1			1			1		1		
Sleep bruxism													
(0–4)	3 (0–4)	2 (0–4)	3 (0.75–4)	**0.055** ^**c**^	3 (0–4)	3 (0–4)	0.668^c^	3 (0–4)	4 (2–4)	**0.004** ^**c**^	3 (0–4)	3 (1–4)	0.793^c^
Awake bruxism													
(0–4)	3 (2–3)	3 (2–3)	3 (2–3)	0.816^c^	2 (0.75–3)	3 (2–3)	**0.156** ^**c**^	3 (2–3)	3 (2–3)	**0.100** ^**c**^	3 (2–3)	3 (2–3)	0.370^c^
Anxiety													
(0–3)	0 (0–1)	0 (0–1)	0 (0–1)	0.895^c^	0 (0–1)	0 (0–1)	0.262^c^	0 (0–1)	0 (0–1)	**0.076** ^**c**^	0 (0–1)	0 (0–1)	0.301^c^
Missing	3	1	2		1	2		2	1		1	2	
Physical symptoms													
(0–3)	1 (0–2)	1 (0–1.5)	1 (1–2)	**0.134** ^**c**^	1 (0–1)	1 (0–2)	0.272^c^	1 (0–1.75)	2 (1–3)	**< 0.001** ^**c**^	1 (0–2)	1 (1–2)	0.765^c^
Missing	2	1	1		1	1		1	1		1	1	
Depression													
(0–4)	0 (0–1)	0 (0–1)	1 (0–1)	**0.178** ^**c**^	0 (0–1)	0 (0–1)	0.547^c^	0 (0–1)	0|1|2	**0.004** ^**c**^	0 (0–1)	0 (0–1)	0.545^c^
Missing	2	1	1		1	1		1	1		1	1	
Perceived stress													
(0–4)	1 (1–2)	1 (1–2)	1 (1–2)	0.998^c^	1 (1–2)	1 (1–2)	0.464^c^	1 (1–2)	1 (1–2)	**0.173** ^**c**^	1 (1–2)	1 (1–2)	0.398^c^
Missing	1		1			1			1		1		
Optimism about effect of treatment													
(1–10)	7 (5–8)	7 (6–8)	7 (5–8)	0.465^c^	6.5 (5–8)	7 (5.75–8)	**0.117** ^**c**^	7 (5–8)	7 (6–8)	0.675^c^	7 (6–8)	7 (5–8)	0.339^c^
Previous treatment				0.883^a^			**0.093** ^**a**^			0.426^a^			0.486^a^
No	84 (60.0)	28 (60.9)	56 (59.6)		14 (46.7)	70 (63.6)		65 (61.9)	19 (54.3)		38 (63.3)	46 (57.5)	
Yes	56 (40.0)	18 (39.1)	38 (40.4)		16 (53.3)	40 (36.4)		40 (38.1)	16 (45.7)		22 (36.7)	34 (42.5)	
Assisted mouth opening				0.257^a^			0.783^b^			0.405^a^			0.893^a^
< 5 mm	112 (80.0)	35 (77.8)	77 (85.6)		25 (86.2)	87 (82.1)		87 (84.5)	25 (78.1)		47 (82.5)	65 (83.3)	
≥ 5 mm	23 (16.4)	10 (22.2)	13 (14.4)		4 (13.8)	19 (17.9)		16 (15.5)	7 (21.9)		10 (17.5)	13 (16.7)	
Missing	5	1	4		1	4		2	3				
**Primary clinician‐related factors**													
Sex				0.724^b^			0.215^b^			1.000^b^			0.518^b^
Female	114 (91.9)	35 (89.7)	79 (92.9)		22 (84.6)	92 (93.9)		87 (91.6)	27 (93.1)		49 (94.2)	65 (90.3)	
Male	10 (8.1)	4 (10.3)	6 (7.1)		4 (15.4)	6 (6.1)		8 (8.4)	2 (6.9)		3 (5.8)	7 (9.7)	
Missing	16	7	9		4	12		10	6		8	8	
Age				0.513^a^			0.447^a^			0.770^a^			0.209^a^
< 30 years	40 (32.3)	11 (28.2)	29 (34.1)		10 (38.5)	30 (30.6)		30 (31.6)	10 (34.5)		20 (38.5)	20 (27.8)	
≥ 30 years	84 (67.7)	28 (71.8)	56 (65.9)		16 (61.5)	68 (69.4)		65 (68.4)	19 (65.5)		32 (61.5)	52 (72.2)	
Missing	16	7	9		4	12		10	6		8	8	
Level				0.663^a^			**0.006** ^**a**^			0.736^a^			0.745^a^
Trainee	76 (61.3)	25 (64.1)	51 (60)		22 (84.6)	54 (55.1)		59 (62.1)	17 (58.6)		31 (59.6)	45 (62.5)	
Staff	48 (38.7)	14 (35.9)	34 (40)		4 (15.4)	44 (44.9)		36 (37.9)	12 (41.4)		21 (40.4)	27 (37.5)	
Missing	16	7	9		4	12		10	6		8	8	
Experience				0.925^a^			**0.066** ^**a**^			0.853^a^			0.906^a^
< 10 years	66 (53.2)	21 (53.8)	45 (52.9)		18 (69.2)	48 (49.0)		51 (53.7)	15 (51.7)		28 (53.8)	38 (52.8)	
≥ 10 years	58 (46.8)	18 (46.2)	40 (47.1)		8 (30.8)	50 (51.0)		44 (46.3)	14 (48.3)		24 (46.2)	34 (47.2)	
Missing	16	7	9		4	12		10	6		8	8	

*Note:* Data are presented as mean ± SD for normally distributed variables; median (Q1‐Q3) for non‐normally distributed variables. *p* values ≤ 0.20 are in bold.

^a^ Chi square test.

^b^ Fischer's exact test.

^c^ Mann–Whitney *U* test.

#### 3.3.1 Occlusal Appliance

Diagnosis, GCPS, sleep bruxism, physical symptoms, and depression were included in the regression analysis due to the *p* values < 0.20 in the univariate analyses (Table 3). Based on the multivariate regression analysis with backward selection, having a DC/TMD pain diagnosis was significantly associated with receiving an occlusal appliance, meaning that a patient with a TMD pain diagnosis is significantly more likely to receive an occlusal appliance compared to a non‐pain diagnosis (Table 4).

**TABLE 4:** Multivariable binary logistic regression analyses for received occlusal appliance (*n* = 138).

Factors	Full model			Model with backward selection		
	β (SE)	OR (95% CI)	*p*	β (SE)	OR (95% CI)	*p*
**Patient‐related factors**						
Diagnosis						
No pain	Reference					
Pain	0.846 (0.433)	2.330 (0.997, 5.443)	0.051	0.954 (0.398)	2.596 (1.189, 5.669)	**0.017**
GCPS	0.167 (0.182)	1.181 (0.827, 1.687)	0.359			
Sleep bruxism	0.189 (0.113)	1.209 (0.969, 1.507)	0.093			
Physical symptoms	0.013 (0.264)	1.014 (0.604, 1.701)	0.959			
Depression	0.114 (0.267)	1.121 (0.664,1.894)	0.669			

*Note: p* values ≤ 0.05 are in bold.

Abbreviations: β, regression coefficient; CI, confidence interval; GCPS, graded chronic pain scale; OR, odds ratio; SE, standard error.

#### 3.3.2 Physical Therapy

Sex, diagnosis, GCPS, awake bruxism, optimism about effect of treatment, previous treatment, clinician's level, and clinician's years of experience were entered in the subsequent regression analysis due to the *p* values < 0.20 in the univariate analyses (Table 3). Based on the multivariate regression analysis with backward selection, received physical therapy was significantly associated with a TMD pain diagnosis (*p* = 0.009), awake bruxism (*p* = 0.015), and clinician's level (*p* = 0.004) (Table 5). More specifically, receiving a DC/TMD pain diagnosis, more frequent awake bruxism and being staff increased the odds of receiving physical therapy as compared to receiving a non pain diagnosis, not having awake bruxism and being trainee.

**TABLE 5:** Multivariate binary logistic regression analyses for received physical therapy (*n* = 123).

Factors	Full model			Model with backward selection		
	β (SE)	OR (95% CI)	*p*	β (SE)	OR (95% CI)	*p*
**Patient‐related factors**						
Sex			0.728			
Female	Reference					
Male	0.223 (0.641)	1.250 (0.356, 4.390)				
Diagnosis			**0.036**			**0.009**
No pain	Reference			Reference		
Pain	1.167 (0.556)	3.213 (1.080, 9.563)		1.355 (0.519)	3.876 (1.401, 10.721)	
GCPS	0.287 (0.236)	1.333 (0.8440, 2.114)	0.223			
Awake bruxism	0.592 (0.238)	1.808 (1.134, 2.884)	**0.013**	0.548 (0.225)	1.730 (1.112, 2.690)	**0.015**
Optimism about effect of treatment	0.229 (0.131)	1.258 (0.973, 1.625)	0.080			
Previous treatment			0.263			
No	Reference					
Yes	−0.589 (0.526)	0.555 (0.198, 1.557)				
**Primary clinician‐related factors**						
Level			**0.016**			**0.004**
Trainee	Reference			Reference		
Staff	2.308 (0.957)	10.051 (1.541, 65.561)		1.803 (0.622)	6.068 (1.729, 20.553)	
Experience						
< 10 years	Reference					
10+ years	−0.595 (0.821)	0.552 (0.110, 2.760)	0.469			

*Note: p* values ≤ 0.05 are in bold.

Abbreviations: β, regression coefficient; CI, confidence interval; OR, odds ratio; SE, standard error.

#### 3.3.3 Psychological Treatment

Diagnosis, GCPS, sleep bruxism, awake bruxism, anxiety, physical symptoms, depression and perceived stress were selected for the subsequent regression analysis due to the *p* values < 0.20 in the univariate analyses (Table 3). Based on the multivariate regression analysis with backward selection, received psychological therapy was significantly associated with a TMD pain diagnosis (*p* = 0.038), sleep bruxism (*p* = 0.025) and physical symptoms (*p* < 0.001) (Table 6). This means that receiving a DC/TMD pain diagnosis, more frequent sleep bruxism and having physical symptoms increased the odds of receiving psychological treatment as compared to receiving a non pain diagnosis, not having awake bruxism and not having physical symptoms.

**TABLE 6:** Multivariateb logistic regression analyses for received psychological treatment (*n* = 137).

Factors	Full model			Model with backward selection		
	β (SE)	OR (95% CI)	*p*	β (SE)	OR (95% CI)	*p*
**Patient‐related factors**						
Diagnosis			**0.047**			**0.038**
No pain	Reference			Reference		
Pain	1.380 (0.696)	3.975 (1.015, 15.561)		1.389 (0.671)	4.013 (1.077, 14.951)	
GCPS	0.061 (0.223)	1.063 (0.687, 1.645)	0.785			
Sleep bruxism	0.375 (0.172)	1.455 (1.039, 2.036)	**0.029**	0.323 (0.144)	1.381 (1.041, 1.830)	**0.025**
Awake bruxism	−0.144 (0.264)	0.866 (0.516, 1.453)	0.586			
Anxiety	0.033 (0.361)	1.034 (0.510, 2.099)	0.926			
Physical symptoms	0.802 (0.308)	2.230 (1.219, 4.081)	**0.009**	0.947 (0.256)	2.578 (1.561, 4.259)	**< 0.001**
Depression	0.211 (0.328)	1.235 (0.649, 2.350)	0.521			
Perceived stress	0.128 (0.350)	1.136 (0.572, 2.256)	0.715			

*Note: p* values ≤ 0.05 are in bold.

Abbreviations: β, regression coefficient; CI, confidence interval; GCPS, graded chronic pain scale; OR, odds ratio; SE, standard error.

#### 3.3.4 Deviation From Indicated Treatment

Only patient's age was selected for the subsequent regression analysis. However, deviation from indicated treatment was not significantly associated with patient's age (β (SE) = −0.014 (0.011), OR (95% CI) = 0.986 (0.965, 1.008), *p* = 0.215) in the multivariate regeression analysis.

## 4 Discussion

This study aimed to investigate the type of treatment TMD patients at the OPD clinic of ACTA actually received compared to the treatment that was indicated at baseline. In addition, the aim was to identify variables in patient and clinician profiles that would predict the type of treatment that patients received and identify variables that would predict possible deviations from the indicated treatment. There was moderate agreement between indicated and received treatment for occlusal appliance, and only fair agreement between indicated and received treatment for physical therapy, psychological therapy, CES and medication. Counselling was received by all participants. Next, physical therapy was the most frequent treatment. Moreover, this study showed that several independent variables, both patient and primary clinician‐related, could predict receiving a specific type of treatment.

Having a DC/TMD pain diagnosis increased the likelihood of receiving occlusal appliance, physical therapy, and psychological therapy, which illustrates that TMD pain management requires multidisciplinary approaches [22]. In the meantime, patients reported higher frequency of awake bruxism increased the chance of receiving physical therapy, whereas sleep bruxism increased the chance of receiving psychological therapy. Since TMD pain and bruxism are both associated with psychosocial factors [23], the presence of awake bruxism may need to be considered when planning physical therapy for TMD pain. In addition, this result may be associated with the myofeedback technique used by our physiotherapists as part of the interventions at ACTA. Myofeedback is mainly indicated in the presence of awake bruxism [24]. In this study, all patients regardless of whether they received psychological treatment exhibited comparable level of awake bruxism. Patients may report high levels of stress or other symptoms that led the clinician to recommend psychological treatment. Although previous studies have found that awake bruxism was more strongly associated with TMD and psychosocial factors such as stress than sleep bruxism [25], patients with SB may also be influenced by neuroticism—a personality trait characterised by negative emotions such as worrying, and associated with stress and anxiety [26]. Thus, the association between sleep bruxism and psychosocial factors should not be overlooked. At ACTA, our strategy is to explain the role of psychological factors to the patients first. This is not unexpected for the patients, because they have already completed a comprehensive set of questionnaires, including psychological screening tools, before their first consultation. When indicated, we then offer the option of a psychological consultation as part of our interdisciplinary team approach.

Being treated by a staff clinician increase the odds of receiving physical therapy. In other words, primary clinicians' differentiation seemed to influence wheter physical therapy was part of the actually received treatment of the participants. Literature describes an association between the clinicians' number of years passed since graduation and their clinical decision‐making in TMD management [10]. Practitioners who graduated more recently indicated a higher TMD treatment need among their patients. It is noted that, at ACTA, trainees may provide basic physical therapy by themselves, whereas staffs may refer patients to a physical therapist due to their tight clinical schedules. This situation, combined with the complexity of TMD pain, might affect the treatment plan. In order to understand how clinician's differentiation level and experience influence deviations from received treatment, testing whether more experienced clinicians would indicate the same treatment as the less experienced ones when looking at the same randomly picked patient profiles might help future studies.

Deviation between indicated and received treatment was observed for more than half of the participants. However, a significant association was found between almost all indicated and actually received treatment modalities. Even though this finding seems contradictory, it might be explained by the relatively high number of participants who did not receive psychological treatment, even though this was indicated at baseline. Moreover, the high deviation rate might be related to the length of the follow‐up period. The sample consisted of 140 participants, with a follow‐up period of maximally 6 months from intake. For all participants receiving treatment that lasted longer than 6 months, the full treatment trajectory was not assessed. In some cases, these observations might lead to a falsely registered deviation between indicated treatment at baseline and actually received treatment. In addition, other factors including patients' financial status, insurance plan or environmental support, such as social support, were not included in the analysis. Extending the follow‐up period and considering other factors for the deviation is a good recommendation for future research in order to investigate longitudinal deviations from indicated treatment at baseline.

To the best of our knowledge, this is the first study to compare originally indicated and actually received TMD treatment, and to identifty potential predictors for the received treatment. No significant predictors for deviation from the indicated treatment at baseline were found. The potential predictors for the received treatment were selected based on previous research and consensus among the investigators, but there might be unidentified factors causing deviation. When screening the patient files, patients rejecting treatment was common. Especially psychological treatment was described as ‘unnecessary’ by patients receiving their indicated treatment plan during intake. Also, the disappearance or significant reduction of complaints after intake may have caused patients to discontinue any further treatment. In some cases, the patient could no longer be reached, resulting in ending treatment for unknown reasons. Moreover, logistic reasons, such as long waiting lists and full schedules at the OPD clinic could have played a role in patients'decisions to discontinue treatment. Future studies which include these types of potential patient‐related predictors might give new insights in treatment deviations.

When indicating a treatment plan, one of the main goals is to develop an effective plan that fits the patient's expectations and the clinic's resources. It is important to consider individual patient‐related factors to estimate whether the patients will actually adhere to the indicated treatment plan. Taking a look at the literature describing conditions that could be compared to TMD, like low back pain, patients' recovery expectations show a strong association with future work participation and a less strong association with recovery outcomes [27]. A study assessing factors possibly associated with physical therapy for shoulder pain also found a high impact of patients expectations and wishes regarding the treatment outcome. This knowledge might be helpful when predicting the success rate for TMD treatment as well and could be taken into consideration when indicating a treatment plan. Also, these variables related to patient expectations can be included as potential predictors in future studies of the assessment of TMD treatment deviations.

Even though this is a retrospective study, the meticulous data collection procedure is considered a strength. Since all of the possible treatment categories were identified very precisely and extensively, it was clear wheter a participant should be assigned to a specific indicated or received treatment, strengthening the validity and reliability of the present study.

Not all clinicians performing intakes for the participants responded to the clinician questionnaire, resulting in absent data assessing the primary clinician‐related factors. When more extensive data from the primary clinicians can be collected, future studies might find significant associations between primary clinician‐related factors and the indicated and actually received treatment. Moreover, a larger sample of clinicians is recommended in future studies. Nevertheless, an association between clinician's years passed since graduation and TMD management has already been reported [10].

Finally, it cannot be ensured that the exact same TMD management strategies are used in other treatment settings and/or in other countries. Since the OPD clinic of ACTA is a highly specialised department with an experienced multidisciplinary team, we assume that we included and evaluated all the sufficient treatment options needed to perform the present study and make it eligible for other multidisciplinary settings treating TMD. The present model provides information on the patient profiles related to the type of received treatment in the OPD clinic and could be informative when setting up a treatment plan in such settings. This may make clinicians decision‐making and guidance during treatment more accurate and effective. Future studies could focus on the decision making process and treatment adherence of TMD patients in other settings, such as general practices, and countries with different healthcare systems.

## 5 Conclusion

A good to perfect agreement between indicated and received treatments was observed for all treatments, except psychological treatment, suggesting that indicated and received TMD treatment are related. Predictors for receiving a specific type of treatment were identified, concluding that receiving an occlusal appliance, physical therapy and psychological treatment were associated with patient‐ and primary clinician‐related factors. No significant predictors were found for deviation, suspecting that unidentified predictors were not included in the present study. Future studies including different variables might give new insights into the assessment of TMD treatment deviations.

## Author Contributions

K.E. and M.T. contributed to conception, design, acquisition, analysis and interpretation, drafted, and critically revised manuscript; N.S. contributed to analysis and interpretation, drafted, and critically revised manuscript; M.K. contributed to data acquisition and critically revised the manuscript. T.C. and F.L. contributed to the conception, design, analysis and interpretation, drafted, and critically revised the manuscript. All authors read and approved the final manuscript.

## Ethics Statement

The study was approved by the ACTA Ethics Committee (ref. No. 2021‐47 483), and followed the principles of the Declaration of Helsinki.

## Conflicts of Interest

The authors declare no conflicts of interest.

## Peer Review

The peer review history for this article is available at https://www.webofscience.com/api/gateway/wos/peer‐review/10.1111/joor.70012.

## Data Availability

The datasets used and/or analysed during the current study are available from the corresponding author on reasonable request.

## References

[1] M. Osiewicz , F. Lobbezoo , B. Ciapala , J. Pytko‐Polonczyk , and D. Manfredini , “Pain Predictors in a Population of Temporomandibular Disorders Patients,” Journal of Clinical Medicine 9, no. 2 (2020): 452. 32041274 10.3390/jcm9020452 PMC7074020

[2] A. Rollman , C. M. Visscher , R. C. Gorter , and M. Naeije , “Care Seeking for Orofacial Pain,” Journal of Orofacial Pain 26, no. 3 (2012): 206–214. 22838005

[3] A. Blanco‐Aguilera , E. Blanco‐Aguilera , R. Serrano‐Del‐Rosal , et al., “Influence of Clinical and Psychological Variables Upon the Oral Health‐Related Quality of Life in Patients With Temporomandibular Disorders,” Medicina Oral, Patología Oral y Cirugía Bucal 22, no. 6 (2017): e669–e678. 29053648 10.4317/medoral.21746 PMC5813984

[4] A. Gil‐Martinez , M. Grande‐Alonso , I. Lopez‐de‐Uralde‐Villanueva , A. Lopez‐Lopez , J. Fernandez‐Carnero , and R. La Touche , “Chronic Temporomandibular Disorders: Disability, Pain Intensity and Fear of Movement,” Journal of Headache and Pain 17, no. 1 (2016): 103. 27812883 10.1186/s10194-016-0690-1 PMC5095086

[5] C. S. Mienna and A. Wanman , “Self‐Reported Impact on Daily Life Activities Related to Temporomandibular Disorders, Headaches, and Neck‐Shoulder Pain Among Women in a Sami Population Living in Northern Sweden,” Journal of Orofacial Pain 26, no. 3 (2012): 215–224. 22838006

[6] R. Ohrbach , E. Bair , R. B. Fillingim , et al., “Clinical Orofacial Characteristics Associated With Risk of First‐Onset TMD: The OPPERA Prospective Cohort Study,” Journal of Pain 14, no. 12 Suppl (2013): T33–T50. 24275222 10.1016/j.jpain.2013.07.018 PMC3855658

[7] L. Baad‐Hansen , M. Thymi , F. Lobbezoo , and P. Svensson , “To What Extent Is Bruxism Associated With Musculoskeletal Signs and Symptoms? A Systematic Review,” Journal of Oral Rehabilitation 46, no. 9 (2019): 845–861. 31090938 10.1111/joor.12821

[8] N. Su , C. M. Visscher , A. J. van Wijk , F. Lobbezoo , and G. J. van der Heijden , “A Prediction Model for Types of Treatment Indicated for Patients With Temporomandibular Disorders,” Journal of Oral & Facial Pain and Headache 33, no. 1 (2019): 25–38. 30129939 10.11607/ofph.2076

[9] A. Gil‐Martinez , A. Paris‐Alemany , I. Lopez‐de‐Uralde‐Villanueva , and R. La Touche , “Management of Pain in Patients With Temporomandibular Disorder (TMD): Challenges and Solutions,” Journal of Pain Research 11 (2018): 571–587. 29588615 10.2147/JPR.S127950 PMC5859913

[10] D. R. Reissmann , A. Behn , O. Schierz , T. List , and G. Heydecke , “Impact of Dentists' Years Since Graduation on Management of Temporomandibular Disorders,” Clinical Oral Investigations 19, no. 9 (2015): 2327–2336. 25843051 10.1007/s00784-015-1459-7

[11] M. Thymi , M. C. Verhoeff , C. M. Visscher , and F. Lobbezoo , “Patient‐Based Experiences With the Use of an Ambulatory Electromyographic Device for the Assessment of Masticatory Muscle Activity During Sleep,” Journal of Oral Rehabilitation 47, no. 5 (2020): 557–566. 32056251 10.1111/joor.12945 PMC7317933

[12] F. Lobbezoo , G. Aarab , M. O. Ahlers , et al., “Consensus‐Based Clinical Guidelines for Ambulatory Electromyography and Contingent Electrical Stimulation in Sleep Bruxism,” Journal of Oral Rehabilitation 47, no. 2 (2020): 164–169. 31430389 10.1111/joor.12876 PMC7004009

[13] A. Zani , F. Lobbezoo , A. Bracci , J. Ahlberg , and D. Manfredini , “Ecological Momentary Assessment and Intervention Principles for the Study of Awake Bruxism Behaviors, Part 1: General Principles and Preliminary Data on Healthy Young Italian Adults,” Frontiers in Neurology 10 (2019): 169. 30881335 10.3389/fneur.2019.00169 PMC6405426

[14] D. R. Reissmann , “Dental Patient‐Reported Outcome Measures Are Essential for Evidence‐Based Prosthetic Dentistry,” Journal of Evidence‐Based Dental Practice 19, no. 1 (2019): 1–6. 30926098 10.1016/j.jebdp.2019.01.003

[15] E. Schiffman , R. Ohrbach , E. Truelove , et al., “Diagnostic Criteria for Temporomandibular Disorders (DC/TMD) for Clinical and Research Applications: Recommendations of the International RDC/TMD Consortium Network* and Orofacial Pain Special Interest Groupdagger,” Journal of Oral & Facial Pain and Headache 28, no. 1 (2014): 6–27. 24482784 10.11607/jop.1151 PMC4478082

[16] S. Cohen , T. Kamarck , and R. Mermelstein , “A Global Measure of Perceived Stress,” Journal of Health and Social Behavior 24, no. 4 (1983): 385–396. 6668417

[17] K. Kroenke , R. L. Spitzer , and J. B. Williams , “The PHQ‐9: Validity of a Brief Depression Severity Measure,” Journal of General Internal Medicine 16, no. 9 (2001): 606–613. 11556941 10.1046/j.1525-1497.2001.016009606.x PMC1495268

[18] K. Kroenke , R. L. Spitzer , and J. B. Williams , “The PHQ‐15: Validity of a New Measure for Evaluating the Severity of Somatic Symptoms,” Psychosomatic Medicine 64, no. 2 (2002): 258–266. 11914441 10.1097/00006842-200203000-00008

[19] R. L. Spitzer , K. Kroenke , J. B. Williams , and B. Lowe , “A Brief Measure for Assessing Generalized Anxiety Disorder: The GAD‐7,” Archives of Internal Medicine 166, no. 10 (2006): 1092–1097. 16717171 10.1001/archinte.166.10.1092

[20] M. J. van der Meulen , F. Lobbezoo , I. H. Aartman , and M. Naeije , “Validity of the Oral Behaviours Checklist: Correlations Between OBC Scores and Intensity of Facial Pain,” Journal of Oral Rehabilitation 41, no. 2 (2014): 115–121. 24274580 10.1111/joor.12114

[21] M. Von Korff , J. Ormel , F. J. Keefe , and S. F. Dworkin , “Grading the Severity of Chronic Pain,” Pain 50, no. 2 (1992): 133–149. 1408309 10.1016/0304-3959(92)90154-4

[22] R. Leeuw , G. D. Klasser , and American Academy of Orofacial Pain , Orofacial Pain: Guidelines for Assessment, Diagnosis, and Management, 6th ed. (Quintessence Publishing Co, Inc., 2018).

[23] L. C. Voss , H. Basedau , P. Svensson , and A. May , “Bruxism, Temporomandibular Disorders, and Headache‐A Narrative Review of Correlations and Causalities,” Pain 165 (2024): 2409–2418. 38888745 10.1097/j.pain.0000000000003277

[24] T. Chattrattrai , M. Thymi , N. Su , and F. Lobbezoo , “Changes in Self‐Reported Sleep and Awake Bruxism in Relation to the Management of Temporomandibular Disorders (‘Care as Usual’) in a Specialty Clinic Population,” Dental and Medical Problems 61, no. 5 (2024): 697–704. 39480974 10.17219/dmp/193125

[25] T. Chattrattrai , G. Aarab , N. Su , T. F. Blanken , S. Mitrirattanakul , and F. Lobbezoo , “The Association of Self‐Reported Awake Bruxism and Sleep Bruxism With Temporomandibular Pain and Dysfunction in Adult Patients With Temporomandibular Disorders,” Clinical Oral Investigations 27 (2023): 7501–7511. 37864603 10.1007/s00784-023-05338-y

[26] T. Strausz , S. Strausz , S. E. Jones , et al., “A Two‐Sample Mendelian Randomization Study of Neuroticism and Sleep Bruxism,” Journal of Dental Research 103, no. 10 (2024): 980–987. 39185608 10.1177/00220345241264749 PMC11409563

[27] J. A. Hayden , M. N. Wilson , R. D. Riley , R. Iles , T. Pincus , and R. Ogilvie , “Individual Recovery Expectations and Prognosis of Outcomes in Non‐Specific Low Back Pain: Prognostic Factor Review,” Cochrane Database of Systematic Reviews 2019, no. 11 (2019): CD011284. 31765487 10.1002/14651858.CD011284.pub2 PMC6877336

